# Comparative Evaluation of Solite RS3 and HyFlex Remover Retreatment Files in Conserving Remaining Dentin Thickness During Endodontic Retreatment Using Cone Beam Computed Tomography: An In Vitro Analysis

**DOI:** 10.7759/cureus.57805

**Published:** 2024-04-08

**Authors:** Anjali Sankar, Pradeep Solete, Ganesh Jeevanandan, Delphine Priscilla Antony, Nishitha Arun, Sandhya Raghu

**Affiliations:** 1 Department of Conservative Dentistry and Endodontics, Saveetha Dental College and Hospitals, Saveetha Institute of Medical and Technical Sciences, Saveetha University, Chennai, IND; 2 Department of Pediatric and Preventive Dentistry, Saveetha Dental College and Hospitals, Saveetha Institute of Medical and Technical Sciences, Saveetha University, Chennai, IND

**Keywords:** retreatment, solite rs3, retrieval, remaining dentin thickness, gutta-percha, novel heat-treated files, innovation, hyflex remover, dental

## Abstract

Introduction

Non-surgical retreatment is seen as a conservative choice for dealing with recurrent infections, instead of opting for periapical surgery. The retreatment processes should be promptly and efficiently carried out, utilizing a suitable armamentarium. The objective of this experiment is to evaluate the quantity of root dentin that remains following the removal of gutta-percha (GP) from the root canal employing two distinct retreatment files.

Materials and methods

Sixty single-rooted teeth were selected for the examination. The process of shaping and cleaning was performed using the step-back approach, with a master apical file size of 40. The smear layer was effectively eliminated by rinsing with a solution of 3% sodium hypochlorite and 17% ethylenediaminetetraacetic acid. Paper points were employed to desiccate the canals. The obturation process involved the utilization of the lateral compaction technique with the AH Plus sealer (Dentsply Sirona, NC, USA). The teeth were classified into two groups: Group I (n=30) underwent retreatment using HyFlex Remover (Coletene India, Pvt., Ltd.), whereas Group II (n=30) received therapy with Solite RS3 retreatment files (Solite Dental in Chennai, India). The remaining dentin thickness (RDT) was assessed by cone beam computed tomography at levels 3, 6, and 9 mm from the cemento enamel junction after the removal of GP. The acquired data underwent examination using an independent t-test to determine statistical significance.

Results

The findings demonstrate that the utilization of Solite RS3 files led to a higher level of dentin thickness remaining at 3 mm, 6 mm, and 9 mm on the mesial side in comparison to HyFlex Remover retreatment files. The observed difference was found to be statistically significant at a significance level of p<0.05 on the mesial side. Nevertheless, there was no notable disparity seen between the two file types at these three levels on the distal side (p>0.05).

Conclusion

Based on the obtained results of the study, it can be concluded that Solite RS3 files show promise in preserving the RDT. However, further studies encompassing diverse parameters are needed to establish a conclusive and definitive conclusion.

## Introduction

The success of root canal therapy is influenced by important parameters such as proper disinfection and instrumentation of the root canal. The instrumentation process directly affects the amount of remaining dentin thickness (RDT), subsequently impacting the root's resistance to fractures [1]. Previous research has indicated that a minimum remaining dentin thickness of 0.3-0.5 mm is necessary to ensure the long-term success of endodontically treated teeth [2]. Preserving a sufficient amount of dentin thickness is essential for increasing the resistance to fracture and the overall strength of the treated teeth.

Preserving the RDT is particularly significant in orthograde retreatment cases [3]. This is due to the potential for procedural errors such as dentin removal and perforation during retreatment procedures [4]. Orthograde retreatment involves removing the existing filling material, thoroughly cleaning and shaping the canal, and then re-obturating it [5]. The success rate of orthograde retreatment, ranging from 74% to 98%, has been reported to be favorable compared to other treatment options [6]. However, the outcome of retreatment is also influenced by the procedural approach utilized.

During the instrumentation process, a significant amount of dentin is typically removed. In previous times, hand files were frequently employed for the retrieval of the obturating material [7]. At first, carbon files were used, but these had the disadvantage of being susceptible to corrosion and tarnishing. As a result, stainless-steel files were adopted [8]. However, stainless steel files were found to be less flexible. To address these limitations, nickel-titanium rotary instruments were introduced. However, achieving an optimal taper while simultaneously preserving the remaining dentin proved to be challenging during root canal preparation [9]. Preserving the residual dentin is especially crucial in circumstances where retreatment is necessary. Therefore, it is crucial to carefully select appropriate files, especially during retreatment procedures [10,11].

The HyFlex Remover retreatment system (Coletene India, Pvt., Ltd.) is frequently used in orthograde retreatment treatments. The Hyflex Remover file system from Switzerland is designed specifically for retreatment procedures. The system is characterized by a single file with a taper of 7% and an apical diameter size of 30. The file is equipped with a 1 mm wire that is minimally intrusive and is recognized for its high efficiency without requiring any solvent. The file is fitted with non-cutting points and is offered in two different lengths: 15.5 mm and 18.5 mm. The 15.5 mm file features a blade that is active solely at its tip, but the 18.5 mm file has a blade that is active along its whole length. Based on the study done by Sowmya et al. [12], the Solite RS3 retreatment file system (Solite Dental in Chennai, India) was chosen for the study. It is a pioneering set of files designed specifically for retreatment procedures. This system comprises three distinct files: RS1, RS2, and RS3, each featuring tapers that are varied in length and cutting points. Notably, the RS2 and RS3 files have been subjected to a heat-treatment process, which augments their flexibility. In contrast, traditional retreatment files frequently result in greater dentin removal when extracting gutta-percha (GP), potentially causing iatrogenic errors. In cone beam computed tomography (CBCT), one can evaluate the tooth in all three dimensions for the amount of remaining dentin and remanent filling material post-treatment by superimposing the image obtained [13]. In contrast, the heat-treated Solite RS3 files offer the advantage of being flexible and capable of efficiently retrieving GP while preserving the surrounding dentin [13-15]. The objective of this study is to evaluate the quantity of root dentin that remains following the removal of GP from the root canal using two distinct retreatment files at depths of 3 mm, 6 mm, and 9 mm. The null hypothesis stated that there is no statistically significant difference found between the two retreatment files while preserving the RDT at all three levels.

## Materials and methods

Specimen preparation

The study involved the selection of 60 mandibular premolar teeth with fully developed apices and periodontal compromises. The teeth were placed in a 5% glutaraldehyde solution. The study gained ethical clearance from the Institutional Ethical Committee (SRB/SDC/ENDO-2080/20/092) to guarantee compliance with ethical principles. In order to qualify for the study, the teeth needed to satisfy specific criteria. These included having only one canal, being undamaged and without any cracks, displaying no evidence of resorption inside or outside the tooth, and having a canal curvature of less than 15°. Digital radiography was used to confirm the presence of a single straight canal in both the buccolingual and mesiodistal directions. The teeth were subsequently decrowned using a diamond disk and adjusted to a specified length of 18 mm.

An access cavity was created, and a glide path was established using a #10 K-file. The canals were shaped and cleaned using the step-back approach, with the master apical file size set at 40 K-file. During the procedure, ample irrigation was performed using 3% NaOCl and 17% EDTA to effectively eliminate inorganic and organic debris from the tooth. Canal drying was facilitated using paper points. The obturation process involved the utilization of AH Plus as a sealer (Dentsply Sirona, NC, USA), using the lateral compaction technique. After the obturation process was completed, CareStream 3D Imaging version V3.10.21 software (Carestream Dental LLC, Mumbai, India) was used for obtaining pre-operative and post-operative CBCT of the specimens. Sample specimens were exposed to 120 KV voltage and 5 mA power for 12 seconds with a dose of 642 mGy.cm2 and voxel size 150 μm x 150 μm x 150 μm in continuous scan mode to assess the dentin thickness, both before and after the removal of GP in the axial section at 3 mm, 6 mm, and 9 mm, respectively.

**Figure 1:**
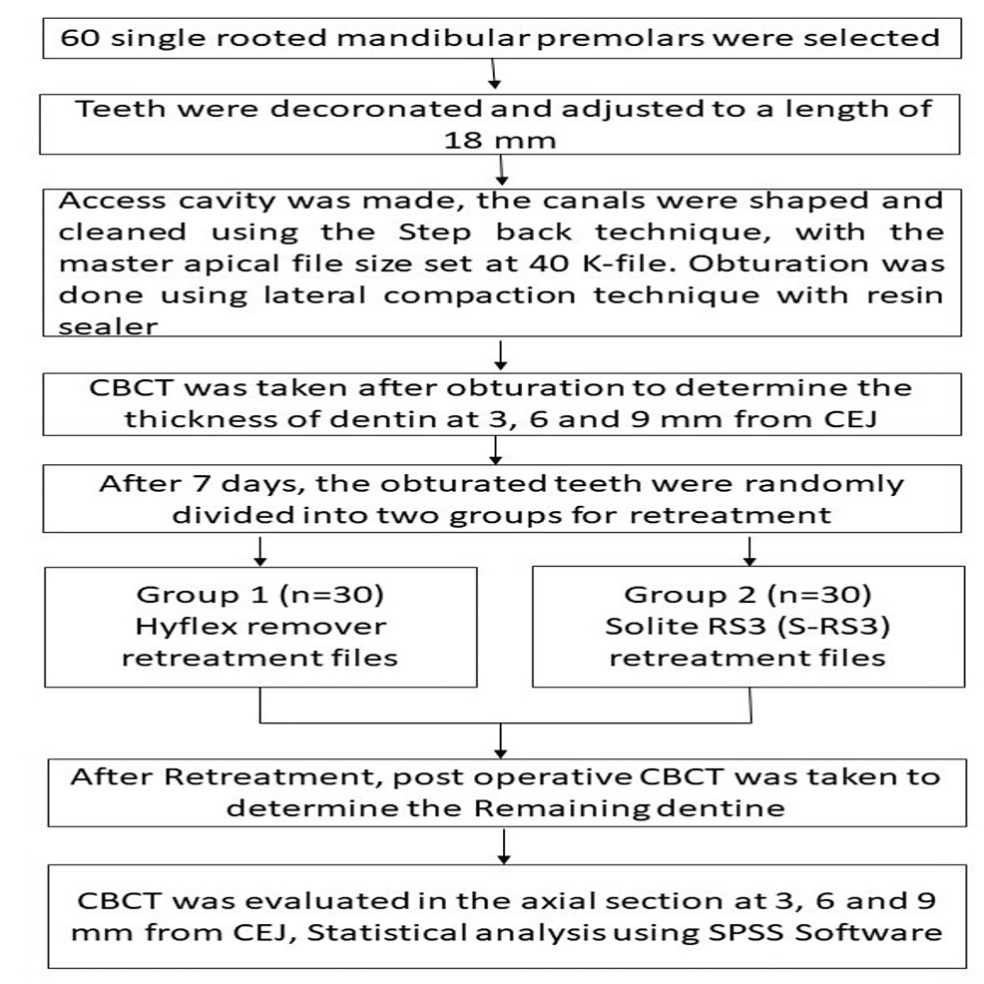
Methodology for specimen preparation and evaluation CEJ: cemento-enamel junction, CBCT: cone beam computed tomography

After a period of seven days, the teeth that had been previously obturated were randomly assigned to two groups using block randomization, which was assisted by the website www.randomization.org. Each of these groups had 30 teeth. These teeth underwent retreatment procedures, employing either the HyFlex Remover retreatment files or the Solite RS3 retreatment files as per the group assignments. Notably, no chemical solvents were utilized during the retreatment process. Throughout the GP removal procedure, the root canals were periodically irrigated with a solution of 3% NaOCl and 17% EDTA, and the final rinse was executed using a saline solution. Following the GP retrieval based on the manufacturing instructions given for the respective files, a second CBCT scan was performed to evaluate the RDT. This study involved four investigators, each assigned to different tasks, including sample preparation, the evaluation of RDT using CBCT, and the subsequent statistical analysis (Figure 1).

RDT evaluation

**Figure 2:**
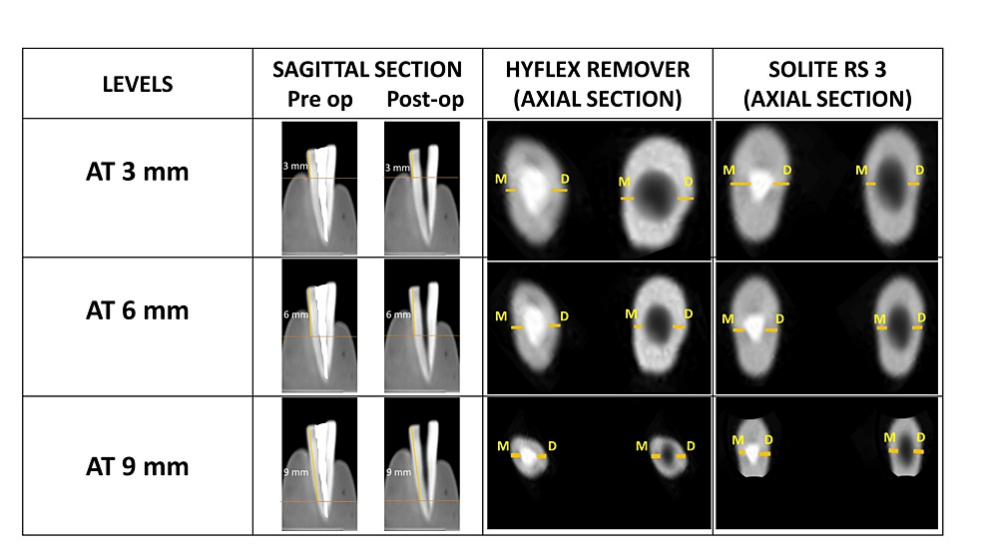
Axial section (RDT) of pre-op and post-op CBCT at 3 mm, 6 mm, and 9 mm for HyFlex Remover and Solite RS3 retreatment files at mesial (M) and distal (D) RDT: remaining dentin thickness, CBCT: cone beam computed tomography

The pre-operative images were initially measured at 3 mm, 6 mm, and 9 mm in the sagittal section, and the slice positioner was moved accordingly. The measurements were recorded in the axial section, both mesial and distal to the canal, respectively. The post-operative values were also recorded based on the same (Figure 2).

Statistical analysis

The gathered data underwent analysis using SPSS Statistics version 23.0 (IBM Corp. Released 2015. IBM SPSS Statistics for Windows, Version 23.0. Armonk, NY: IBM Corp.). A t-test was performed at intervals of 3 mm, 6 mm, and 9 mm from the cemento-enamel junction (CEJ) in both mesial and distal orientations. The significance of the results was determined using a statistical significance level of p<0.05.

## Results

**Figure 3:**
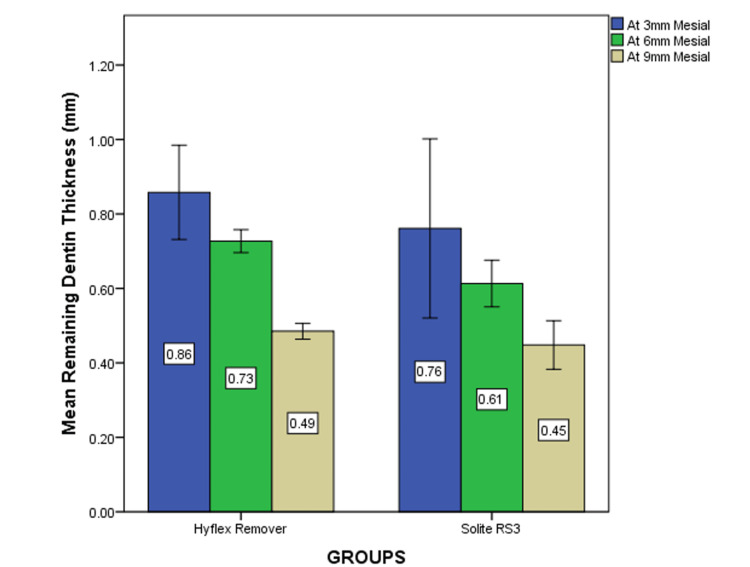
The mean remaining dentin thickness at the mesial side: x-axis blue represents the RDT at 3 mm, green represents the RDT at 6 mm, and beige represents the RDT at 9 mm. Statistical difference was seen at all three levels for Solite RS3 when compared to HyFlex Remover retreatment files (p<0.05) RDT: remaining dentin thickness

**Figure 4:**
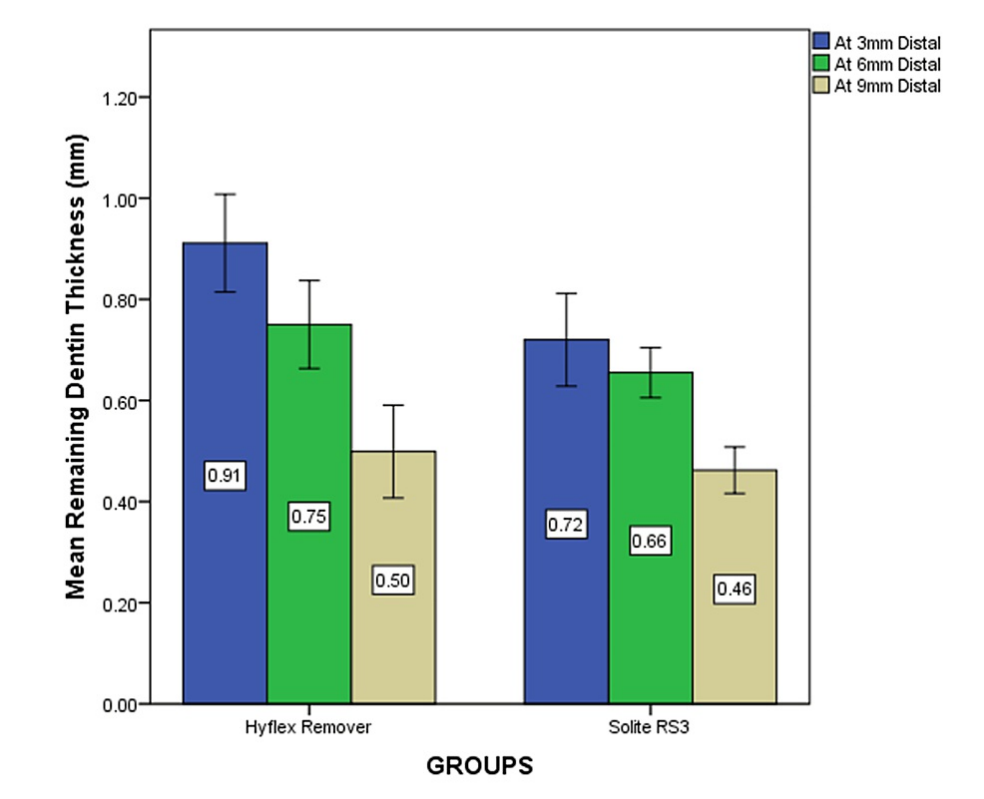
The mean remaining dentin thickness at the distal side: x-axis blue represents the RDT at 3 mm, green represents the RDT at 6 mm, and beige represents the RDT at 9 mm. Statistical difference was not seen at all three levels for Solite RS3 when compared to HyFlex Remover retreatment files (p>0.05) RDT: remaining dentin thickness

The mean and standard deviation values for the RDT at the mesial and distal surfaces of the CEJ were evaluated at 3 mm, 6 mm, and 9 mm between the Solite RS3 and HyFlex Remover. The independent t-test revealed a statistically significant difference at all three levels (p<0.05). At 3 mm (p=0.014), 6 mm (p=0.034), and 9 mm (p=0.029) (Table 1 and Figure 3). On the distal side, the amount of dentin removed by the Solite RS3 group is less compared to the HyFlex Remover group. However, the independent t-test indicated there was no statistically significant difference observed at any of the three levels (p>0.05). At 3 mm (p=0.791), 6 mm (p=0.121), and 9 mm (p=0.232), respectively (Table 2 and Figure 4).

**Table 1:** Mean and standard deviation values for the RDT at the mesial surface The results show the comparative mean and standard deviation values for the RDT at the mesial surface from the CEJ at 3 mm, 6 mm, and 9 mm between Solite RS3 and HyFlex Remover. Independent t-tests showed a statistically significant difference at all three levels (p<0.05): at 3 mm (p=0.014), 6 mm (p=0.034), and 9 mm (p=0.029) RDT: remaining dentin thickness, CEJ: cemento-enamel junction

Levels	Groups	Mean ± Std. deviation	p-value
At 3 mm mesial	HyFlex Remover	0.8580 ± 0.12665	0.014
	Solite RS3	0.7610 ± 0.24058	
At 6 mm mesial	HyFlex Remover	0.7270 ± 0 .03093	0.034
	Solite RS3	0.6130 ± 0.06255	
At 9 mm mesial	HyFlex Remover	0.4850 ± 0.02121	0.029
	Solite RS3	0.4480 ± 0 .06512	

**Table 2:** Mean and standard deviation values for the RDT at the distal surface The results show the comparative mean and standard deviation values for the RDT at the distal surface from the CEJ at 3 mm, 6 mm, and 9 mm between Solite RS3 and HyFlex Remover. An independent t-test showed no statistically significant difference at all three levels (p>0.05): at 3 mm (p=0.791), 6 mm (p=0.121), and 9 mm (p=0.232), respectively RDT: remaining dentin thickness, CEJ: cemento-enamel junction

Levels	Groups	Mean ± Std. deviation	p-value
At 3 mm distal	HyFlex Remover	0.9110 ± 0.09678	0.791
	Solite RS3	0.7200 ± 0.09189	
At 6 mm distal	HyFlex Remover	0.7500 ± 0.08705	0.121
	Solite RS3	0.6550 ± 0.04972	
At 9 mm distal	HyFlex Remover	0.4990 ± 0.09195	0.232
	Solite RS3	0.4620 ± 0.04590	

## Discussion

The efficacy of endodontic therapy hinges on three pivotal factors: meticulous irrigation, efficient disinfection, and comprehensive closure of the root canals [16]. Out of these procedures, cleaning and shaping are regarded as the most crucial in root canal operations since they directly influence the thickness of the dentin [17]. It is essential to adequately prepare the canal to facilitate the flow of irrigants and ensure proper obstruction of the root canal [18]. Care must be taken during shaping and cleaning to avoid over-enlarging the canals, as this could compromise the dentin thickness. The amount of remaining dentin directly affects the fracture resistance of endodontically treated teeth, which, in turn, influences the long-term survival of the tooth [19]. Hence, preserving sufficient dentin is vital for the structural integrity and longevity of the tooth. Research has indicated that most dentin removal occurs during mechanical instrumentation, particularly in the mesial and distal directions during retreatment procedures [20]. Therefore, clinicians must exercise caution and precision during these stages to maintain an adequate dentin thickness while effectively cleaning and shaping the root canal system.

Achieving an appropriate canal taper is essential, as it facilitates the proper flow of irrigants into the complex areas of the root canal system [1]. During retreatment procedures, caution must be exercised during instrumentation, as it immediately impacts the thickness of the remaining root dentin, which might have already been compromised during the initial root canal procedure [21]. Therefore, selecting the appropriate files becomes crucial to effectively remove the obturating material while preserving the dentin thickness.

Several previous studies have conducted comparisons to assess the efficacy of different file systems in the removal of obturating material [22,23]. These experiments adhered to particular protocols, which involved the utilization of chemical solvents to extract GP and the implementation of two-dimensional digital radiography to verify the thorough removal of the filling material. Nevertheless, the present investigation chose to abstain from using solvents due to apprehensions about potential discrepancies in the findings. Prior studies have shown that solvents can result in substantial residues of endodontic material and sealer on the walls of the root canal during retreatment procedures [24,25]. Moreover, the use of solvents may lead to further irritation of periapical tissues, making it challenging to accurately determine the retrieval time, as reported in previous studies.

Aktuna Belgın et al. conducted a study that determined that traditional radiography lacks precision in accurately representing the thickness of dentin and may overstate its real measurement [26]. To precisely assess the remaining obturating material, the study suggests employing different techniques, including CBCT, microcomputed CT, and longitudinal sectioning. For the present study, CBCT was chosen as the preferred method to assess the RDT. The CBCT provides a comprehensive evaluation of the prepared canal space in three dimensions, allowing for more accurate measurements [27]. The study focused on three specific areas within the root canal system: 3 mm, 6 mm, and 9 mm from the CEJ. These locations represent the coronal, middle, and apical thirds of the root canal, where there is a higher likelihood of iatrogenic errors occurring during root canal procedures. Using CBCT to evaluate dentin thickness in these areas provides valuable insights for assessing the success and safety of the obturation process.

In this study, the Solite RS3 file system was found to result in less dentin removal compared to the HyFlex Remover retreatment files. This outcome is attributed to the fact that Solite RS3 files are heat-treated, which provides them with increased flexibility. This flexibility allows them to effectively remove the obturating material without causing damage to the root dentin. The Solite RS3 file possesses alternate cutting edges, which facilitate the simpler elimination of the obturating material. Additionally, their extraordinary flexibility acts as a protective measure against the excessive removal of dentin. On the other hand, the HyFlex Remover retreatment files, which also have alternate cutting edges and lack radial lands, are effective in retrieving obturating material. However, due to their 7% taper, they can inadvertently affect the root dentin during the procedure [17].

The study focused on single-rooted teeth with an oval cross-sectional shape, being wider from front to back (mesiodistally) and narrower from side to side (buccolingually). Owing to anatomical reasons, the shaping and filling of the root canals tend to be more in the mesial aspect compared to the distal aspect; this can be attributed to the circular root canal preparation by the rotary files. When using rotary files with a round cross-section, excessive dentin removal occurs in the mesial rather than the distal directions. These findings are in agreement with the study done by Sowmya et al. [12], Shahriari et al. [28], and Limongi et al. [29]. Unlike other files, the HyFlex Remover retreatment files have a modified triangular cross-sectional shape. This shape has three different cutting edges and does not have any radial lands. Consequently, these files are specifically engineered to prevent penetration into the canal wall and efficiently eliminate excess dentin removal. The study was limited to the analysis of single-rooted teeth. Further investigations on multirooted teeth and comparison with other retreatment file systems might be valuable from a clinical perspective.

## Conclusions

The study's results indicate that utilizing Solite RS3 files holds the potential for maintaining dentin thickness during retreatment procedures, which is an essential factor to consider at all three levels. The null hypothesis showed a statistically significant difference between the two retreatment files while preserving the RDT at all three levels in the mesial aspect. In the distal aspect, Solite RS3 preserved more dentin; however, it was not statistically significant. Nevertheless, it is imperative to highlight the necessity for further investigation to examine other aspects and variables that might influence the effectiveness of retreatment. These include alternative techniques for root canal filling, using solvents, and evaluating various retreatment tools. To obtain more definitive conclusions, future studies should encompass a wider range of parameters and consider conducting clinical trials to verify the findings from laboratory testing.
